# Silicone-Textile Composite Resistive Strain Sensors for Human Motion-Related Parameters

**DOI:** 10.3390/s22103954

**Published:** 2022-05-23

**Authors:** Joshua Di Tocco, Daniela Lo Presti, Alberto Rainer, Emiliano Schena, Carlo Massaroni

**Affiliations:** 1 Unit of Measurements and Biomedical Instrumentation, Departmental Faculty of Engineering, Università Campus Bio-Medico di Roma, Via Alvaro del Portillo, 00128 Rome, Italy; j.ditocco@unicampus.it (J.D.T.); d.lopresti@unicampus.it (D.L.P.); c.massaroni@unicampus.it (C.M.); 2 Unit of Tissue Engineering and Chemistry for Engineering, Departmental Faculty of Engineering, Università Campus Bio-Medico di Roma, Via Alvaro del Portillo, 00128 Rome, Italy; a.rainer@unicampus.it; 3 Institute of Nanotechnology, National Research Council, Via Monteroni, 73100 Lecce, Italy

**Keywords:** flexible sensors, polymer encapsulation, sensors for physiological monitoring, strain sensors, wearable sensors

## Abstract

In recent years, soft and flexible strain sensors have found application in wearable devices for monitoring human motion and physiological parameters. Conductive textile-based sensors are good candidates for developing these sensors. However, their robust electro-mechanical connection and susceptibility to environmental factors are still an open challenge to date. In this work, the manufacturing process of a silicone-textile composite resistive strain sensor based on a conductive resistive textile encapsulated into a dual-layer of silicone rubber is reported. In the working range typical of biomedical applications (up to 50%), the proposed flexible, skin-safe and moisture resistant strain sensor exhibited high sensitivity (gauge factor of −1.1), low hysteresis (maximum hysteresis error 3.2%) and ease of shaping in custom designs through a facile manufacturing process. To test the developed flexible sensor, two applicative scenarios covering the whole working range have been considered: the recording of the chest wall expansion during respiratory activity and the capture of the elbow flexion/extension movements.

## 1. Introduction

The use of soft and biocompatible sensors to manufacture wearable devices to monitor physiological parameters [1,2,3,4,5,6] and human motion [7,8,9,10,11] is gaining momentum. Soft sensors result in being a valuable option when comfortability and ease of integration are a priority. Resistive [12,13,14,15] and capacitive [16,17,18] sensors have gained broad usage due to their compatibility with materials and their easy readout. Conductive textiles have been proven to be suitable for developing conductive sensors for the mentioned valuable features. Generally, these sensors are obtained from the combination of conductive sensing elements and a polymeric matrix. Common conductive materials involve nanofabrics [19,20,21], conductive fabrics [11,22,23,24], conductive polymers [25,26,27], and carbon nanotubes [28,29,30]. However, if in contact with moisture (e.g., sweat, ambient humidity), the bare textile shows a degradation or failure of the electro-mechanical properties, making it unusable. The encapsulation into a polymeric matrix is potentially helpful in enhancing the robustness and wearability of the sensors, ensuring their reliability over time. To date, many flexible strain sensors have been developed with either high deformation range or high sensitivity, thus limiting their application. As an example, a graphene-based strain sensor showed a high Gauge Factor (*GF*) up to 10^4^ within the strain range of 2–8% [8]. On the other hand, a carbon-nanotube-based sensor with wide strain range (280%) showed a very low *GF* (0.06) [31]. There is a need for a cost-effective and facile manufacturing method for strain sensors with a good trade-off between sensitivity and strain range. To assess the suitability of strain sensors for the application of interest, the performances are evaluated via electro-mechanical testing, analyzing stretchability, linearity, sensitivity, hysteresis, response time and drift [22,32]. The main challenges for these types of sensors are represented by robust electro-mechanical connections that at the same time do not permanently fix the sensor to the support (e.g., sewing, gluing), and by the susceptibility to external influencing factors that may cause a decay of the sensor electro-mechanical properties. Silicone matrices have been used to isolate the sensing elements in order to prevent the influence of external factors, but their integration remains a challenge [1,8,9]. To address these limitations, we report the manufacturing process of a silicone-textile composite resistive strain sensor based on encapsulating a conductive resistive textile into a dual-layer of silicone elastomer. The proposed solution resulted in a flexible, skin-safe and moisture-resistant strain sensor with a set of electro-mechanical properties suitable for biomedical applications at different strain ranges. Indeed, the sensor exhibits a wide stretchability (strain up to 250%) and showed a *GF* of −1.1 in the range of interest (i.e., 0–50%) for monitoring important physiological activities such as breathing and some motions of human joints (e.g., flexion/extension of the elbow joint).

## 2. Materials and Methods

### 2.1. Sensors Manufacturing

Sensors were produced by hand cutting eight rounded rectangle shapes of dimensions (LxW) 120 mm × 15 mm, from an A4 sheet of Eeontex LG-SLPA (Eeonyx Corp., Pinole, CA 94564, USA). Sensors were cut in pairs in course and wale directions of the fabric. Two metallic snaps with a diameter of 11 mm (by Koh-i-Noor Hardmuth, Budějovice, Czechia) were positioned at the ends of the sensor to provide it with a sturdy mechanical fixing and a stable connection to read-out electronics.

**Figure 1:**
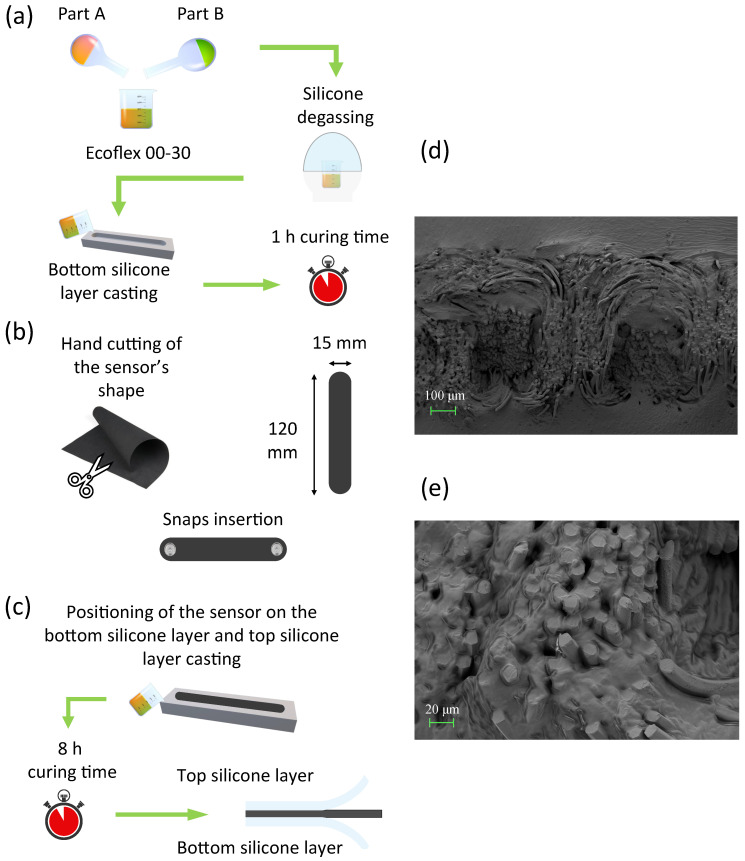
(**a**–**c**) Fabrication route. (**a**) Preparation of the silicone elastomer by mixing the two components, followed by degassing and casting of the bottom silicone layer. (**b**) Cutting of the resistive element and insertion of the metal snaps to endow the sensor with electromechanical connections. (**c**) Positioning of the sensor on the pre-cured bottom silicone layer and casting of the top layer. (**d**) SEM micrograph of the dual layer interface showing the silicone elastomer matrix positively infiltrating the textile. (**e**) SEM micrograph at higher magnification in the same region of interest.

Two pairs of sensors (one course- and one wale-cut) were encapsulated in a dual-layer silicone elastomer matrix. We manufactured the flexible strain sensor by combining a conductive resistive textile with a dual-layer silicone elastomer encapsulation, as illustrated in Figure 1. Silicone elastomer was obtained by mixing SmoothOn Ecoflex 00-30 part A and part B in a 1:1 weight ratio, followed by vacuum-degassing at 860 kPa and room temperature curing for approximately 2 min. A total of 8 g were obtained. First, the bottom layer of silicone was cast and cured into a 3D-printed mould at room temperature for 1 h (Figure 1a). The bare conductive textile was cut in a rectangular shape with rounded corners. Two metallic snaps (11 mm diameter) were installed at its ends (Figure 1b). Finally, the sensor with the metallic snaps was positioned on the bottom silicone layer; the top layer was cast on the textile and cured at room temperature for 8 h obtaining the flexible strain sensor (Figure 1c). The sensor exhibited a resistance change after the encapsulation (from ∼111 kΩ to ∼227 kΩ) due to the infiltration of silicone matrix among the textile fibers, as shown in Figure 1d,e. The microarchitecture of the silicone-encapsulated sensors was characterized by scanning electron microscopy (SEM). Sensor cross-sections were achieved through manual cuts with a razor blade and regions of interest were identified under a Zeiss Axio Zoom v16 stereo microscope. Specimens were then coated with a thin film of sputtered gold and observed under a Zeiss SIGMA 300 VP field emission gun SEM at an acceleration voltage of 5 kV.

### 2.2. Characterization of the Electro-Mechanical Properties

**Figure 2:**
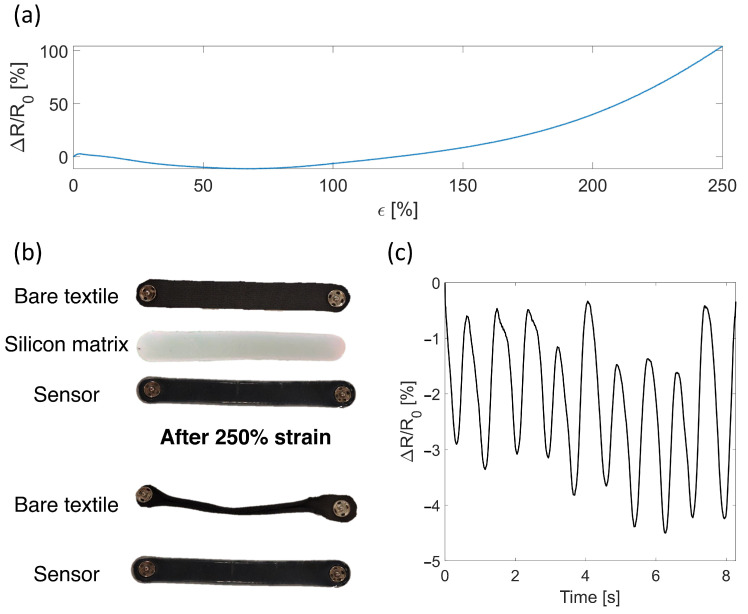
(**a**) Output of the sensors when strained at 250%. (**b**) Photo showing the different sensors tested in the working range assessment and how the silicone matrix enhances the robustness of the sensor and avoids the side rolling of the textile. (**c**) output of the sensor when after 250% strain while performing 10 hand strains.

Mechanical properties were tested on a universal tensile tester (Instron 3365A) equipped with a 500 N load cell providing the force applied to the sample and an encoder providing the crosshead position. Screw side action tensile grips were used to hold the sample. Resistance was estimated from the voltage sensor output by using a custom voltage divider. A commercial data acquisition board (NI-DAQ 6002, National Instruments) was used to both power the circuit at +5 V and to collect the mentioned voltage signal. Both mechanical (i.e., force and displacement applied to the sensors) and electrical data (voltage output of the sensors) were collected at 100 Hz. Electro-mechanical properties have been assessed for all sensors: two course-cut (S1) and two wale-cut (S2) bare textiles, and two course-cut (S3) and two wale-cut (S4) encapsulated textiles. The sensor’s working range was assessed by imposing a 250% strain at 0.028 s^−1^ and its functionality was tested by performing 10 hand strains (Figure 2a,b). Sensors’ sensitivity was assessed by imposing a 50% strain level at 0.05 min^−1^ over five trials. Average and relative expanded uncertainty were calculated.

Sensitivity to strain was assessed by calculating the sensor’s *GF* as follows:

(1) $$GF=\frac{\Delta R/R_{0}}{\epsilon },$$

where $\Delta R/R_{0}$ is the relative resistance change caused by strain and ϵ is the maximum applied strain.

**Figure 3:**
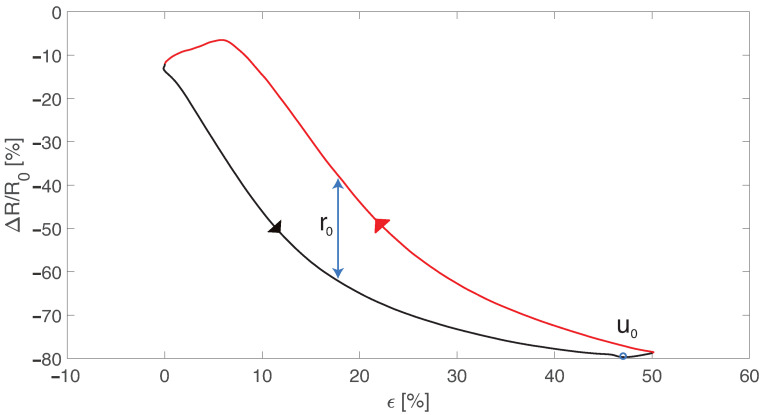
Hysteresis cycle showing the loading (black curve) and the unloading (red curve). $r_{0}$ is the maximum difference between the loading and unloading part of the cycle and $u_{0}$ is the maximum resistance value.

The sensor’s electrical hysteresis was assessed by applying cyclic loading and unloading at 50% strain at 0.028 s^−1^, 0.056 s^−1^, and 0.083 s^−1^. For each speed the hysteresis error was calculated using the following equation:

(2) $$Hyst_{err}=\frac{r_{0}}{u_{0}}\cdot 100,$$

where $r_{0}$ is the maximum difference (in terms of relative resistance change) between the loading and unloading part of the hysteresis cycle and $u_{0}$ is the maximum resistance value. A graphical illustration of the calculation of this error is shown in Figure 3.

We determined the dependence from different strain rates by applying cyclic stretching up to 50% strain at a rate of 0.028 s^−1^, 0.056 s^−1^, and 0.083 s^−1^.

Resistance drift of the sensor was assessed by maintaining strain levels (10%, 20%, 30%, 40%, 50%) for 120 s and evaluating the relative change in resistance.

### 2.3. Moisture Influence on Electrical Resistance

The influence of moisture on the sensor response was assessed at zero strain by investigating the relative resistance changes to different relative humidity levels. This analysis was performed on a bare and an encapsulated sensor. Sensors were positioned in a sealed box at constant temperature (in the range 25.7 ± 0.5 °C considering the whole experiment) and two humidity tests were performed: (i) relative humidity was varied from ∼10% to ∼98% and then brought back to ∼10% and (ii) relative humidity was kept at ∼100% for over 3 h. Temperature and relative humidity within the sealed box were continuously monitored by a thermistor (EL-USB-TP-LCD, LASCAR electronics, Whiteparish) and a capacitive humidity sensor (HIH 4000-002, Honeywell, Padova, Italia) while recording the output of the bare and the encapsulated sensors.

### 2.4. Wearable Prototypes: Manufacturing and Feasibility Assessment in Real Scenarios

To assess the potentiality of the developed flexible sensor, two scenarios have been considered: human motion activity (covering the whole working range) and respiratory activity (low strain). Two wearable devices were instrumented by embedding the proposed sensor for monitoring the respiratory activity and the elbow flexion/extension and were tested on one healthy male volunteer. The elbow support consisted of an elastic elbow guard, while the respiratory monitoring support was an elastic belt with Velcro straps to be adaptable to the torso with different anthropometries. Both supports were endowed with a pair of metallic snaps for both mechanically fixing the sensor on the guard/belt and for connecting it to the read-out electronics. The read-out electronics was composed of a voltage divider and a commercial data acquisition board (NI-DAQ 6002, National Instruments) used to both power the circuit at +5 V and collect the sensor output. The electrical connection of the sensor to the read-out electronics was achieved by threading one end of conductive copper wires in the support’s metal snaps eyelets and by soldering the other to the electronics. All the tests were carried out in compliance with the Ethical Approvals (09/19 OSS ComEt UCBM) and with the written informed consent from the participant. To monitor the respiratory activity, the sensor was placed at the center of an elastic belt provided with metal snaps for easy placement of the sensor and with Velcro strips to make it adaptable to different anthropometries. The wearable elbow monitoring device was tested on a healthy male volunteer in terms of sensitivity to different input angles and of estimation of the number of movements performed. The volunteer was asked to wear the elbow guard with the sensor on top of the elbow joint and to perform two trials: (i) different angles of flexion extension from 0° to 90° with 15° steps and returning to 0° after each step and (ii) 10 full range of motion flexion extensions using the end first one as reference starting point (i.e., a total of 9 total flexion/extensions). All trials were performed at self-paced speed. The wearable respiratory device was tested on a healthy male volunteer in terms of estimation of the respiratory frequency associated with each respiratory regime. The volunteer was asked to wear the belt around the thorax on the xiphoid process and to perform two respiratory trials: (i) Eupnea trial in which the volunteer breathed self-paced in quiet breathing (i.e., ∼0.2 Hz, 12 breaths-per-minute) and (ii) tachypnea trial in which the volunteer breathed self-paced simulating high frequency ventilation (i.e., ∼0.75 Hz, 45 breaths-per-minute).

## 3. Results and Discussion

### 3.1. Electro-Mechanical Properties

The sensor’s stretchability was assessed up to 250% strain to evaluate any occurrence of any destructive phenomenon. Although the bare textiles maintained electrical conductivity at 250% strain, destructive phenomena occurred in reaching this level of strain. As displayed in Figure 2b, the bare textile showed a visible change in shape and size due to the high strain provided with consequent mechanical failure. On the other hand, the encapsulated sensor maintained both the electrical and mechanical properties. As testified by Figure 2c, the encapsulated sensor was able to identify the provided 10 hand strains.

**Figure 4:**
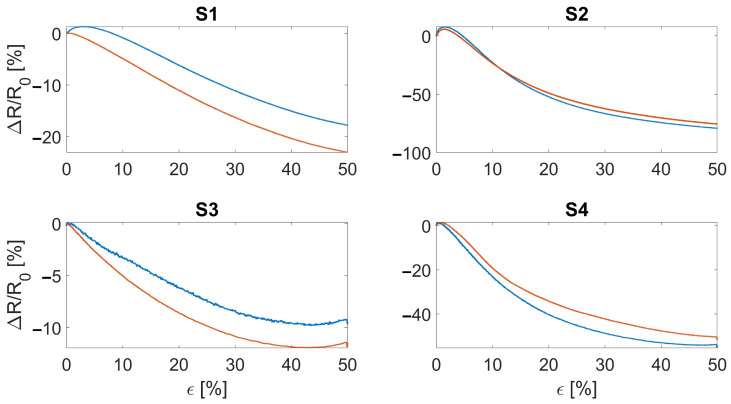
Subplots showing the relative resistance change of the manufactured sensors as a function of the applied strain. S1 and S2 refer to the bare textile cut in the course and wale direction, respectively. S3 and S4 refer to the bare textile cut in the course and wale direction encapsulated into the silicone matrix, respectively. The blue and red curves represent the two manufactured batches.

Considering the sensor’s sensitivity, the wale-cut sensors showed from 20% to 40% higher values in terms of $\Delta R/R_{0}$ compared to the course-cut sensors, as shown in Figure 4. The $\Delta R/R_{0}$ trends indicate a monotonic decreasing resistance to the applied strain. This decreasing monotonic trend is maintained up to ∼70% strain and then changes to a monotonic increasing trend. Despite this exhibited behavior, the sensor is able to identify the strain level from the $\Delta R/R_{0}$ value for all the applications of interest (i.e., respiratory and elbow joint monitoring): indeed, in these applications the developed sensor works within the strain range of 0–50% that shows a monotonic decrease. Moreover, a higher intra-batch difference can be seen in course-cut sensors compared to the wale-cut ones.

**Figure 5:**
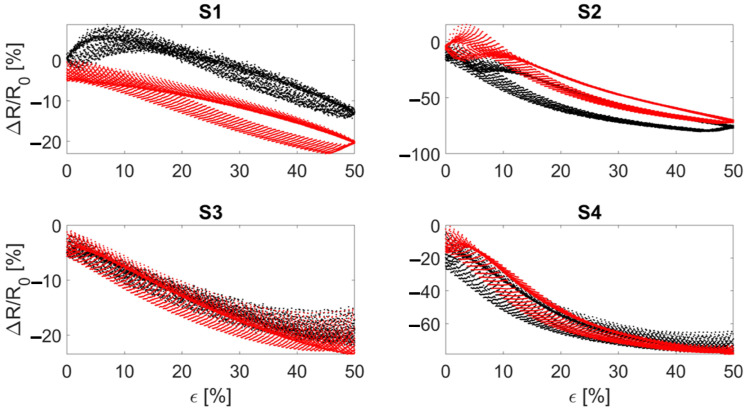
An example of electrical hysteresis of the developed sensors strained consecutively at 0.028 s^−1^. S1 and S2 refer to the bare textile cut in the course and wale direction, respectively. S3 and S4 refer to the bare textile cut in the course and wale direction encapsulated into the silicone matrix, respectively. The black and red curves represent the two manufactured batches.

**Figure 6:**
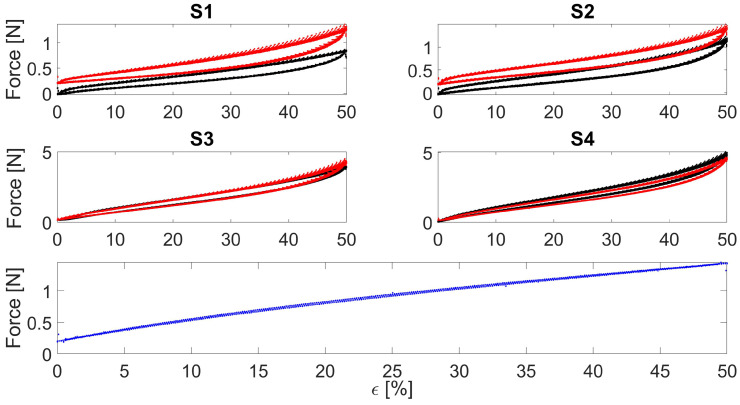
An example of mechanical hysteresis of the developed sensors strained consecutively at 0.028 s^−1^. S1 and S2 refer to the bare textile cut in the course and wale direction, respectively. S3 and S4 refer to the bare textile cut in the course and wale direction encapsulated into the silicone matrix, respectively. The black and red curves represent the two manufactured batches. The bottom blue curve shows the mechanical hysteresis of the silicone matrix alone.

Electrical hysteresis has been evaluated at three different speeds (i.e., 0.028 s^−1^, 0.056 s^−1^, and 0.083 s^−1^) by imposing 50% strain. As a result, both the encapsulated sensors showed lower $Hyst_{err}$ than the bare textiles considering all three speeds: the mean $Hyst_{err}$ values considering the three speeds were 3.2% and 23.2% for the encapsulated and the bare, respectively. In Figure 5 the electrical hysteresis plots at 0.028 s^−1^ are shown. It can be seen that S3 and S4 show overlapping trends among the batches compared to S1 and S2, underlining the lower hysteresis and the benefits obtained from the encapsulation. Indeed, considering Figure 6, this effect can also be seen considering the force as a function of strain where the encapsulated sensors (S3 and S4) show closer loading and releasing curves than the bare ones (S1 and S2) highlighting a lower mechanical hysteresis.

**Figure 7:**
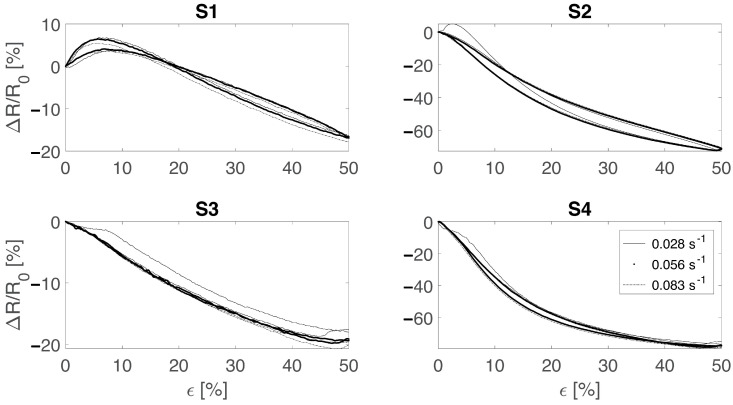
Strain rate influence on the sensors’ output tested at 0.028 s^−1^ (black continuous line), 0.056 s^−1^(black dots), and 0.083 s^−1^ (dashed line). S1 and S2 refer to the bare textile cut in the course and wale direction, respectively. S3 and S4 refer to the bare textile cut in the course and wale direction encapsulated into the silicone matrix, respectively.

Dependence from different strain rates was assessed by applying cyclic stretching up to 50% strain at a rate of 0.028 s^−1^, 0.056 s^−1^, and 0.083 s^−1^ (reported in Figure 7). As a result, the sensors did not show any major dependency from the strain rate. Indeed, except for S1 all sensors showed the same monotonic response to the applied strain with a steeper slope for the wale-cut sensors (S2 and S4). This aspect is in agreement with the sensor’s sensitivity curves (reported in Figure 4) and indicates that the sensitivity is independent from the strain speed. Moreover, as expected from the previous tests, the encapsulated sensors showed a more reliable output when cyclically strained.

**Figure 8:**
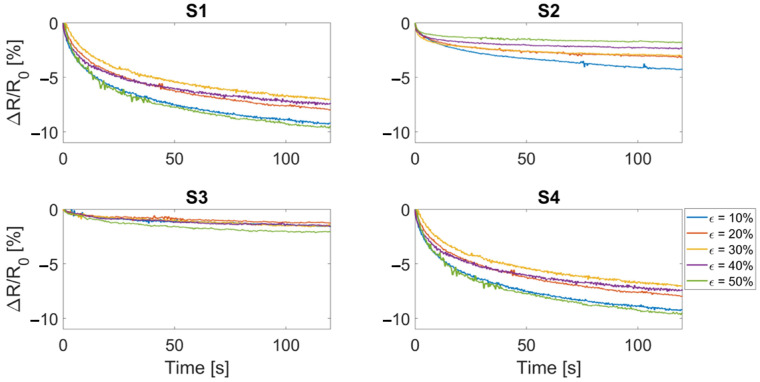
Resistance drift after a strain level was provided and held for 120 s. S1 and S2 refer to the bare textile cut in the course and wale direction, respectively. S3 and S4 refer to the bare textile cut in the course and wale direction encapsulated into the silicone matrix, respectively. Each color represents a strain level.

The resistance drifting under the static strain level was also measured. Drift errors were calculated as the relative resistance change in response to a constant value of strain held for 120 s (reported in Figure 8). Over this time period all sensors showed a resistance drift ranging from a minimum of approximately 2% to a maximum of approximately 10%. This might be a problem for applications which require a static measurement of the displacement. Instead, in the applications of interest (i.e., respiratory monitoring, joint motion monitoring) the most used conditions are cyclic or dynamic.

**Figure 9:**
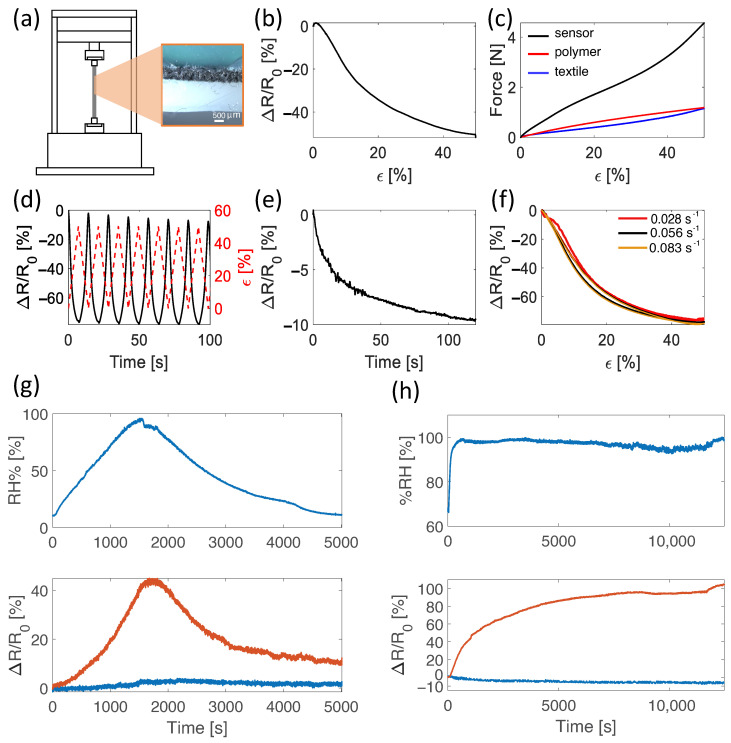
(**a**) Diagram of electro-mechanical test setup and cross-sectional stereophotogrammetric microscope image of the sensor. (**b**) Relative resistance change to 50% strain in quasi-static condition at 0.05 min^−1^. (**c**) Force trend during straining at 50% of the textile alone, the polymeric matrix alone, and the silicone-encapsulated textile (reported as sensor). (**d**) Relative resistance change to triangular cyclic straining to 50% at a speed of 0.056 s^−1^. (**e**) Relative resistance drift when 50% strain is maintained for 120 s. (**f**) Relative resistance change when strained to 50% at 0.028 s^−1^, 0.056 s^−1^ and 0.083 s^−1^. (**g**) Moisture influence on the sensor’s output. Relative humidity provided to the sensors is shown in the top plot. The bottom plot shows changes in relative resistance of the encapsulated sensor (blue line) and the bare sensor (red line). (**h**) Relative resistance changes of the sensor’s when exposed to 100% relative humidity for over 3 h. Relative humidity provided to the sensors is shown in the top plot. The bottom plot shows changes in relative resistance of the encapsulated sensor (blue line) and the bare sensor (red line).

A schematic representation of the electro-mechanical testing setup is shown in Figure 9a along with a cross-sectional stereophotogrammetric microscope image showing the dual layer structure of the manufactured sensor. Considering all the tested electro-mechanical properties, the wale-cut sensor (S4, hereinafter referred to as flexible sensor) displayed the best performances in terms of sensitivity, electrical and mechanical hysteresis error, strain rate dependency and resistance drift. A summary of the mainly relevant metrological properties of the sensors (i.e., *GF*, sensitivity, and hysteresis error) are reported in Table 1.

Specifically, the flexible sensor exhibited good electro-mechanical properties when undergoing 50% strain in quasi-static conditions (i.e., strained at a low speed of 0.05 min^−1^, Figure 9b) with a *GF* of −1.1 in the whole working range 0–50%. This value corresponded to a sensitivity of −3.16 kΩ·%^−1^ and a relative resistance change of −54%. The negative value of the sensitivity is due to the working principle of the sensor, which reduces its resistance with increasing strain. The initial resistance of the sensor was ∼227 kΩ. The bare textile and the polymer showed a similar force value (i.e., ∼1.19 N) to reach a strain level of 50%. When combined, the resulting sensor showed an increase of the mentioned force value from 1.19 N to 4.66 N, as shown in Figure 9c. The cross-section area of the bare textile, the silicone matrix and the encapsulated sensor are approximately 15 mm^2^, 48 mm^2^, and 64 mm^2^, respectively. Calculating the stress from the forces reported in Figure 9c, the stress experienced by the sensor is between the one of the textile and the one of the polymer. Thus, also the elastic modulus of the sensor lies between those of the textile and the matrix. The developed sensor showed output stability when undergoing cyclic stretching up to 50% at all tested speeds (i.e., 0.028 s^−1^, 0.056 s^−1^, and 0.083 s^−1^). Figure 9d shows an example of the cyclic load pattern and the corresponding output at 0.056 s^−1^. The sensor showed an average maximum hysteresis error among all tested speeds of 3.21%, significantly lower than the others. The resistance drifting under static strain level was also measured. Drift errors were calculated as the relative resistance change in response to a constant value of strain (Figure 9e) and were found to be 9.50%, 8.14%, 7.01%, 7.72%, and 9.40% for strain levels of 10%, 20%, 30%, 40%, and 50%, respectively. The strain rate influence on the sensor’s output was also assessed. As shown in Figure 9f, when the sensor was strained to 50% at 0.028 s^−1^, 0.056 s^−1^, and 0.083 s^−1^, no major changes in the output of the sensor were observed, highlighting the sensor reliability over different straining speeds, making it suitable for various applications in the strain range of 0–50%.

The reported high sensitivity along with the low hysteresis demonstrates the promising electrical properties of the sensor. In addition, the high resistance value allows the development of a low power consuming sensor for an improved long-term monitoring scenario without performance decay. In the literature, comparable values have been found in terms of *GF* (i.e., 1.23 and 1.75), hysteresis (i.e., 2.50%) and stretchability (i.e., 150%). Nevertheless, most of the literature focuses on manufacturing a high *GF* sensor and assessing its stretchability, while neglecting other relevant metrological properties (e.g., hysteresis, influence of moisture, output drift, and strain rate). To achieve such high *GF* values, the manufacturing process is quite expensive since the machinery used is highly priced and the whole manufacturing process may be hard to repeat. It is worth noting that there are sensors with outstanding performances in terms of *GF* (i.e., 87 or 535) or stretchability (i.e., 500%) that find their use in various applications but lack the assessment of other metrological properties. However, depending on the application of interest, other characteristics such as high hysteresis may be as important as *GF* value. A summary of the evaluated metrological parameters, manufacturing process and monitored parameters of the literature is reported in Table 2. Concerning the mechanical properties, the sensor showed lower hysteresis compared to the bare textile and polymer, highlighting how the encapsulation process results in a more reliable and more accurate sensor with a more stable and reliable output compared to the bare textile. Most sensors presented in the literature are directly fixed on the skin without any support using medical-like tape or regular tape. This method requires additional time to prepare the sensor for performing the measurement and allows the sensor to be use in only a specific position and for specific application. To provide a multiparametric monitoring sensor applicable to different scenarios, we designed the sensor with metal snaps which make it: (i) easy to fix on a support, (ii) easy to replace in case of failure, and (iii) usable for monitoring multiple parameters by simply changing the support.

### 3.2. Moisture Influence on Electrical Resistance

In the absence of strain, variations of relative humidity in the range ∼10% to ∼98% caused a change in the relative resistance value of the bare textile of ∼45% compared to ∼2% of the encapsulated one (Figure 9g). In addition, when the sensors were kept at ∼100% relative humidity for a long time (i.e., over 3 h), a change in the relative resistance value of ∼100% was observed for the bare textile compared to ∼5% for the encapsulated one (Figure 9h). From these tests, the silicone encapsulation proved to be a good strategy to preserve the electro-mechanical properties of the sensor and to also enable its use in applications in which moisture could be a limiting factor.

### 3.3. Feasibility Assessment of the Wearable System in Real Scenarios: Respiratory and Joint Motion Monitoring

**Figure 10:**
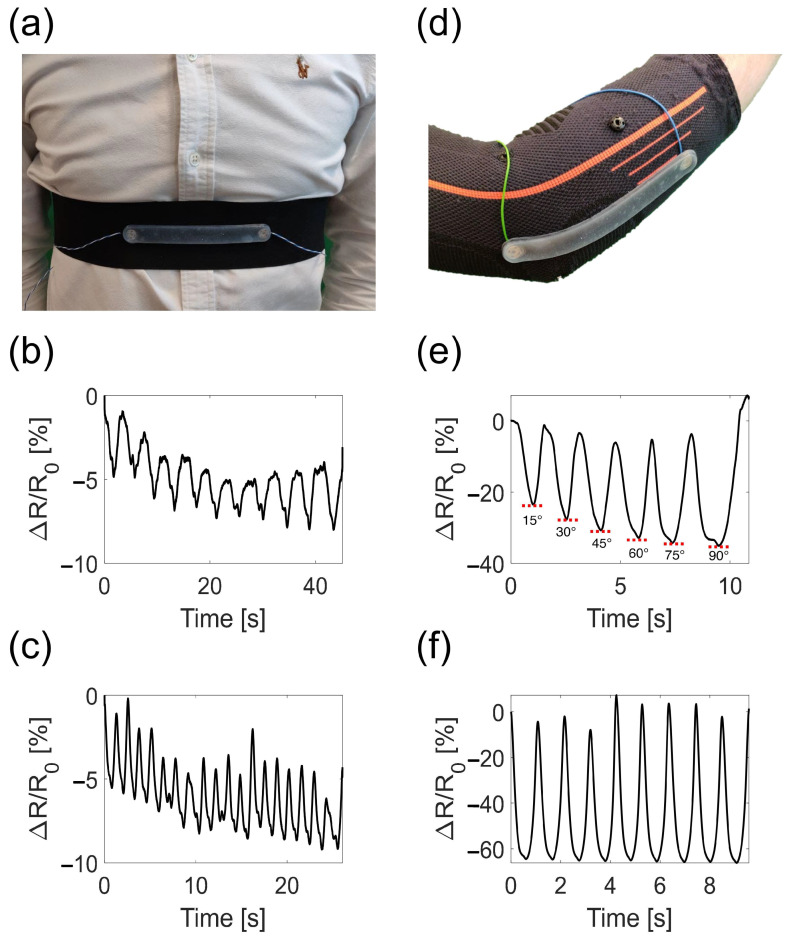
(**a**) Image of the respiratory monitoring device worn by the volunteer at the level of the chest. (**b**) Respiratory trend in the eupnea trial retrieved by the respiratory monitoring device. (**c**) Respiratory trend in the tachypnea trial retrieved by the respiratory monitoring device. (**d**) Image of the elbow monitoring device worn by the volunteer. (**e**) Elbow motion trend retrieved by the elbow monitoring device when different angles at 15° step are provided as input by the volunteer. (**f**) Elbow motion trend retrieved by the elbow monitoring device when 9 full range of motion flexion/extensions are performed.

To monitor the respiratory activity, the sensor was placed at the center of an elastic belt provided with metal snaps for easy placement of the sensor and with Velcro strips to make it adaptable to different anthropometries (Figure 10a). The respiratory monitoring device could identify the different respiratory regimes allowing us to extrapolate the respiratory frequency of the volunteer, as shown in Figure 10b,c. In addition, inspiratory and expiratory peaks were visible and were represented by minima and maxima, respectively, thus allowing us to estimate the respiratory frequency from the inspiratory peaks. As a result, the volunteer breathed at 12 breaths-per-minute (12 bpm, which corresponds to 0.20 Hz) in the eupnea trial and at 45 bpm (which corresponds to 0.75 Hz) in the tachypnea trial. It can be noted that the device output showed a slight drift towards lower resistance values and this may be related to a slight drift of the sensor’s output combined with the volunteer not breathing with the same tidal volume. This aspect is negligible in the proposed application but may be relevant in other applications (for instance, if the wearable is used to estimate respiratory volumes). To monitor the elbow joint flexion/extension, the sensor was placed on top of the elbow joint as shown in Figure 10d. The device could identify the different elbow angles provided as an input. This is clearly visible from the device output signal shown in Figure 10e, where each minimum represents the maximum flexion reached. The increasing angle of the elbow due to flexion caused the device output to decrease accordingly and vice versa. In addition, the number of movements performed by the volunteer could be estimated, taking into consideration the trend shown in Figure 10f and by counting the number of either minima or maxima. Although the maximum value of resistance obtained from the maximum flexion is approximately constant, a slight change in the device output was observed for the full extension (i.e., the starting position, 0°).

## 4. Conclusions

We report the manufacturing process of a silicone-textile composite resistive strain sensor based on the combination of a polymeric matrix and a conductive textile for recording some physiological and physical parameters. The sensor can be fabricated with a cost-effective and facile manufacturing process apt for shape and size customization.

The developed sensor exhibits key advantages when compared to other state-of-the-art technologies: (i) it can be easily manufactured in any shape making it adaptable to various applications; (ii) it overcomes one of the main influencing factors (i.e., moisture) when working with human-related parameters by directly measuring them on the body preventing the decay of the sensor electro-mechanical properties; (iii) it exhibited a good compromise in terms of sensitivity (i.e., *GF* equal to −1.1) and low hysteresis (i.e., 3.2%) within 0–50% strain range and showed good performances considering all the tested properties; (iv) it can be used to monitor different human-related parameters and can be easily removed in the case of failure or for washing the support thanks to the fixing mechanism based on metal snaps.

We demonstrated that the developed sensor is well performing in monitoring both respiratory activity and the human joint motion (in case of elbow flexion/extension) by testing its performance when embedded in wearable supports. We foresee further improving the wearable devices to obtain more integrated and less obtrusive devices with embedded electronics endowed with wireless data transmission. Furthermore, we will perform additional experimental tests on the sensor to assess its dependence from temperature and the moisture influence when undergoing strain. Another important aspect for these sensors is the stability of the metrological properties. Although the silicon encapsulation may prevent/minimize, over a long period of time, the sensor from being subject to any degrading phenomenon, this analysis will deserve more attention before application of the sensor in a real scenario. Finally, we will assess the performances of different shaped sensors to be used for monitoring other physiological parameters with the aim of creating a body-area-network for a complete monitoring device.

## Figures and Tables

**Table 1:** Metrological parameters comparison among the different types of sensors tested.

	*GF* [·]	Sensitivity [kΩ·%−1]	Hysteresis Error [%]
S1	−0.4	−0.1	22.5
S2	−1.6	−1.6	11.2
S3	−0.2	−0.1	10.9
S4	−1.1	−3.2	3.2

**Table 2:** Summarizing table of the main metrological parameters, manufacturing cost, and monitored parameters of the state-of-the-art sensors.

	Sensitivity	*GF*	Hysteresis	Manufacturing	Monitored Parameters	Moisture Influence	Stretchability
[19]	-	7.26 (60%)	R degradation 0.03	Expensive	Vocal vowels	-	150.2%
[20]	114 nF·kPa^−1^	-	-	Expensive	Pressure	-	
[22]	-	1.23	2.50%	Moderate/ Expensive	Finger motion	-	150%
[23]	-	-	-	Moderate/ Expensive	Respiration	-	
[27]	-	87 (ϵ < 40%), 6 (40% < ϵ < 100%)	-	Low-cost/ Moderate	Vocal vowels, pressure	-	100%
[28]	-	1.75	-	Low-cost/ Moderate	Joint motion	-	500%
[29]	-	535	-	Moderate/ Expensive	Respiration	-	150%

## Data Availability

The data presented in this study are available on request from the corresponding author. The data are not publicly available due to privacy reasons.

## Abbreviations

The following abbreviations are used in this manuscript:

GF	Gauge factor
SEM	Scanning electron microscopy
BPM	Breaths per minute
